# Revealing Cycling‐Induced Evolution of Intact Sodium Metal Battery Interfaces Using Cryo‐Focused Ion Beam Cross‐Sectioning and Electron Microscopy

**DOI:** 10.1002/smll.202508531

**Published:** 2025-12-15

**Authors:** Kevin C. Matthews, Rinish R. Vaidyula, Charles B. Mullins, Jamie H. Warner

**Affiliations:** ^1^ Materials Science and Engineering Program and Texas Materials Institute The University of Texas at Austin 204 East Dean Keeton Street Austin TX 78712 USA; ^2^ Walker Department of Mechanical Engineering The University of Texas at Austin 204 East Dean Keeton Street Austin TX 78712 USA; ^3^ Department of Chemistry The University of Texas at Austin Austin TX 78712 USA; ^4^ John J. McKetta Department of Chemical Engineering The University of Texas at Austin Austin TX 78712 USA

**Keywords:** batteries, cryo‐FIB, electron microscopy, materials characterization

## Abstract

Batteries consist of complex, layered interfaces, and their performance‐limiting mechanisms are best understood through nanoscale structural analysis of both anodes and cathodes in realistic full‐cell architectures. This has been challenging for liquid‐electrolyte‐based batteries due to limitations imposed by handling liquid electrolytes and size constraints in most high‐resolution electron microscopes, while cryogenic focused ion beam (cryo‐FIB) milling has typically been limited to a single electrode. Here, a full‐cell cryo‐FIB milling process is presented that reveals anode, cathode, and seprator interfaces in a   liquid electrolyte cell with a sodium metal anode and Na_0.44_MnO_2_ cathode. This full‐cell cryo‐milled battery stack enables visualization of interfaces at both electrodes, allowing characterization of the entire cell while comparing the effects of two solvents, ethlyene carbonat/diethyl carbonate and digylme,  in a NaPF_6_ salt‐based electrolyte. It is demonstrated that after moderate cycling (10–50 cycles), degradation pathways differ between carbonate‐ and ether‐based electrolytes. Carbonate‐based cells degrade rapidly, driven largely by electrolyte depletion resulting from excessive solid electrolyte interphase (SEI) formation at the anode. In contrast, diglyme‐based exhibit improved cycling stability but ultimately also experience electrolyte depletion, which instead arises from electrolyte degradation at the cathode. These findings provide insight into solvent‐specific degradation mechanisms relevant to future battery development.

## Graphical abstract

Cryogenic focused ion beam (cryo‐FIB) cross‐sectioning enables direct visualization of intact sodium metal batteries, revealing solvent‐dependent interfacial degradation pathways. Carbonate‐based electrolytes induce rapid anode failure through excessive solid electrolyte interphase (SEI) formation, while a diglyme‐based electrolyte stabilizes the anode but promotes cathode‐side void formation. This full‐cell cryo‐FIB approach provides mechanistic insight into electrolyte‐driven degradation in Na metal systems.

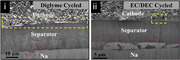

## 1 Introduction

The global transition toward sustainable energy systems necessitates the development of cost‐effective, high‐performance energy storage technologies. Sodium‐ion batteries (SIBs) have emerged as a promising alternative to lithium‐ion batteries (LIBs), primarily due to the abundant and wide‐spread availability of Na.^[^ ^1^ ^]^ SIB precursors (such as Na_2_CO_3_) remain much more affordable than those of LIBs.^[^ ^2^ ^]^ This cost advantage is coupled with similar electrochemical properties to lithium, making Na‐based batteries attractive for large‐scale applications, including grid storage and electric vehicles. Na metal batteries (SMB) are of particular interest due to Na's low reduction potential of −2.71 V vs the standard hydrogen electrode (compared to −3.04 V for Li) and a large theoretical gravimetric energy density of 1166 mAh g^−1^.^[^ ^3^ ^]^ However, the realization of SMBs hinges on identifying suitable electrolytes and cathode materials capable of delivering high capacity, long cycle life, and stability under operational conditions.

The development of SMBs has gained significant attention as a potential alternative to LIBs due to the abundance and low cost of Na resources. However, the search for an appropriate electrolyte for SMBs has proven to be particularly challenging. While many electrolytes studied are direct analogs to those found in LIBs, such as XFP_6_ or XTFSI in carbonate solvents (where X is Na or Li), Na metal exhibits higher reactivity compared to Li, resulting in the formation of a less compact and less robust solid electrolyte interphase (SEI).^[^ ^1^ ^]^

The SEI formed with these conventional electrolytes has been shown to be soluble, leading to continuous dissolution and preventing adequate passivation of the electrode surface.^[^ ^4^ ^]^ This ongoing SEI formation process reduces cycling lifetime and efficiency. In response to these challenges, electrolytes using ether‐based solvents like monoglyme, diglyme, and tetraglyme have demonstrated improved cycling performance.^[^ ^1^, ^5^, ^6^ ^]^ These improvements are attributed to the formation of a more ideal SEI, which limits parasitic reactions and promotes uniform stripping and plating of Na metal.^[^ ^5^ ^]^ Although diglyme has a wide electrochemical stability range, it has been shown to have lower oxidative stability than some carbonate solvents, such as ethylene carbonate (EC).^[^ ^6^ ^]^ Recent complementary studies further underscore the importance of electrolyte–interface coupling in SMBs.^[^ ^7^, ^8^ ^]^

On the cathode side, Na manganese oxides (NMO), a family of layered and tunnel‐structured compounds, have emerged as promising candidates. Their advantages, including high specific capacity, relatively low cost, high structural stability in air, and low environmental impact, make them well‐suited for integration into SMB systems.^[^ ^9^ ^]^ However, while many studies have focused on improving cathode performance through synthesis and doping, little attention has been given to the effects of organic electrolytes on cathode stability.^[^ ^9^, ^10^, ^11^, ^12^, ^13^, ^14^, ^15^ ^]^

Studying the microstructural evolution of the SEI, deposited Na metal, and cathode materials presents significant challenges due to the high reactivity of Na and the SEI, as well as the need to deconstruct the electrochemical cell, which can lead to unintended physical damage. To address these issues, cryogenic sample preparation and cross‐sectional milling techniques have been developed.^[^ ^16^, ^17^ ^]^ These methods allow for the characterization of intact battery cells in their intrinsic state by freezing the cells and preserving solid‐liquid interfaces for electron microscopy analysis.^[^ ^18^ ^]^ This approach minimizes beam‐induced degradation during milling and imaging.^[^ ^19^ ^]^

However, much of this work still involves deconstructing the electrochemical cell, undoubtedly leading to unwanted physical damage to the interfaces. Harrison et al., and Jungjohann et al. demonstrated that intact Li coin cells could be frozen and cross‐sectioned using laser ablation and plasma FIB.^[^ ^20^, ^21^, ^22^ ^]^ By keeping the electrode:separator:electrode stack intact, new insights into interfacial evolution could be gathered. Recently, we showed that a similar process could be applied using only a gallium‐ion FIB to study Na metal electrochemical cells.^[^ ^23^ ^]^ By utilizing a new cryo‐sample preparation and transfer method, we successfully cross‐sectioned liquid electrochemical cells while keeping the electrode/separator/electrode stack intact, avoiding unwanted mechanical deformation from cell dismantling. However, this process has not yet been applied to full battery cells with a standard cathode and a Na metal anode to see how their respective interfaces evolve during cycling.

Given these advancements and the need for a comprehensive understanding of electrolyte effects on SMB performance, this study aims to identify and understand how electrolyte solvents, specifically carbonate (EC: DEC) and glymes (diglyme), impact the microstructural evolution of the SEI deposited Na, and cathode during cycling. To achieve this, cells constructed with Na metal anodes, Na_0.44_MnO_2_ cathodes, and NaPF_6_ in either EC/DEC or diglyme solvent were cycled, subsequently frozen, and cross‐sectioned to study the evolution of the entire cell after cycling. This approach allows for a detailed examination of the interfaces and structures within the battery, providing valuable insights into the complex interplay between electrolyte composition and battery performance in SMBs.

## 2 Results and Discussion

**Figure 1:**
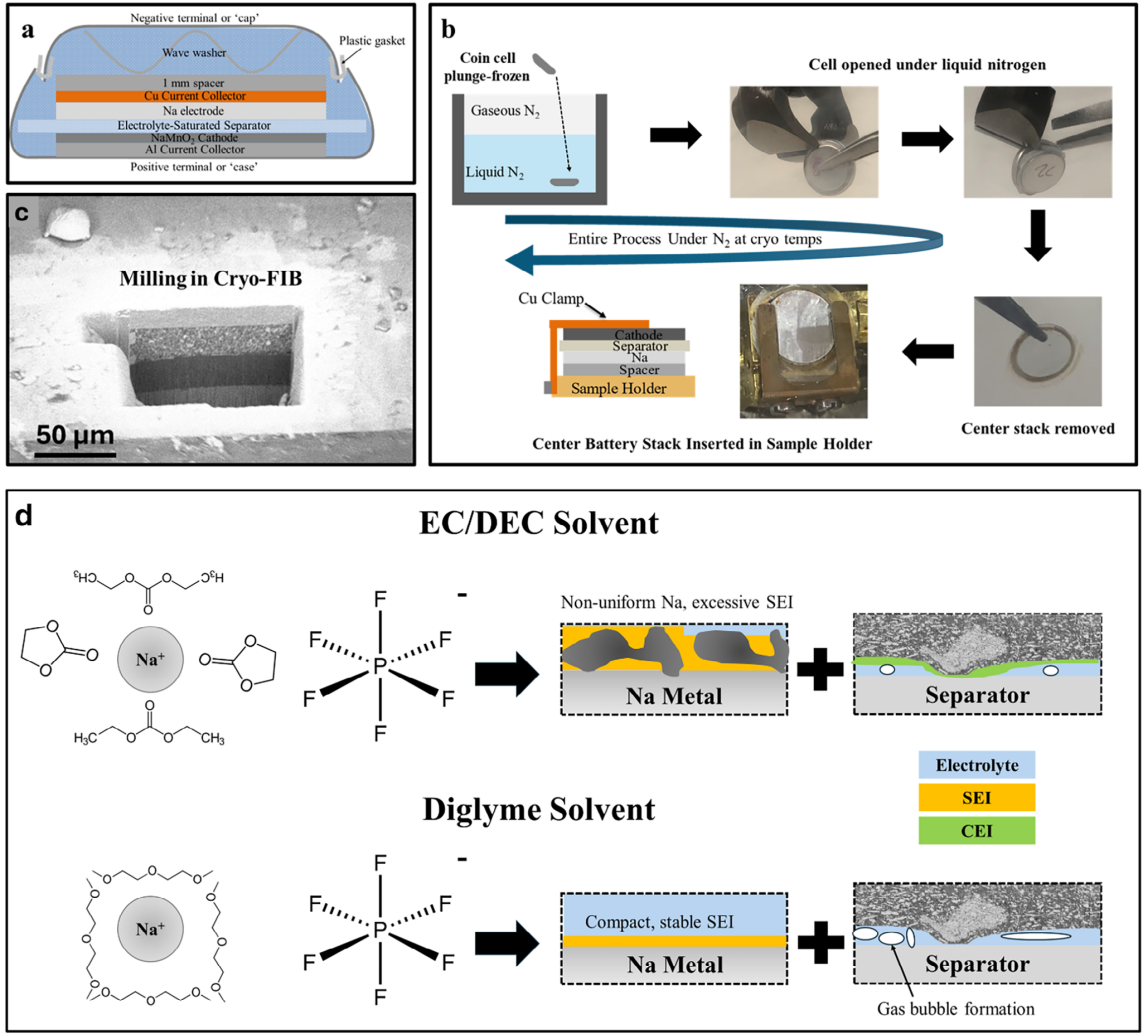
a) Schematic of a Na||NMO coin cell, b) Schematic of the cryo‐sample preparation workflow, c) SEM micrograph following cross‐sectional milling of the sample revealing the cathode, separator, and Na metal anode, d) Schematic describing the expected effects of different solvents on anode and cathode after cycling.

Na metal batteries were constructed using Na_0.44_MnO_2_ as the cathode, and NaPF_6_ in either EC/DEC or Diglyme was used as the electrolyte. **Figure** **1**a shows a schematic of the constructed cells. Cells were plunge‐frozen in liquid nitrogen following the procedure depicted in Figure 1b. Prior results have shown that liquid nitrogen provided an adequate cooling rate for these electrolytes.^[^ ^23^ ^]^ Once frozen, cells were opened using pliers. In our experience, shorting is not a concern at cryogenic temperatures as the kinetics are sufficiently limited essentially preventing any reactions from occurring. Once opened, the outer casing is removed, and the center stack of the electrodes and separator is extracted intact. This stack is placed in the sample holder and retracted into the vacuum‐cryo transfer shuttle (VCT). Throughout the entire process, the cell remains frozen and either in a liquid or gaseous nitrogen environment, thereby never warming nor being exposed to the ambient atmosphere. Even if the sample surface were to react with the ambient environment, the regions of interest are located within the intact stack of battery components and, therefore, never exposed until after milling.

Milling with a gallium ion beam allows for cross‐sectional imaging of the three layers (cathode, separator, and anode), with their respective interfaces preserved, as shown in Figure 1c. This allows the effect of the electrolyte solvent (a carbonate‐based EC/DEC mixture or the ether‐based diglyme) on the evolution of the cell's microstructure after cycling to be studied, as schematically illustrated in Figure 1d. Carbonate solvents, such as the 1:1 volume ratio mixture of EC/DEC we use, are known to promote excessive SEI formation rich in Na_2_CO_3_ and Na‐organic species, which are soluble in the electrolyte. Ether‐based solvents, such as diglyme, are known to produce a more compact SEI that is richer in inorganic compounds like NaF and Na_2_O. These compounds are less soluble in the electrolyte. However, diglyme has been shown to have a lower oxidative stability than EC/DEC, suggesting that it may be more susceptible to side reactions and gas evolution when the cell is charged to high voltages. These side reactions and gas evolution are expected to contribute to void formation at the cathode‐separator interface.

**Figure 2:**
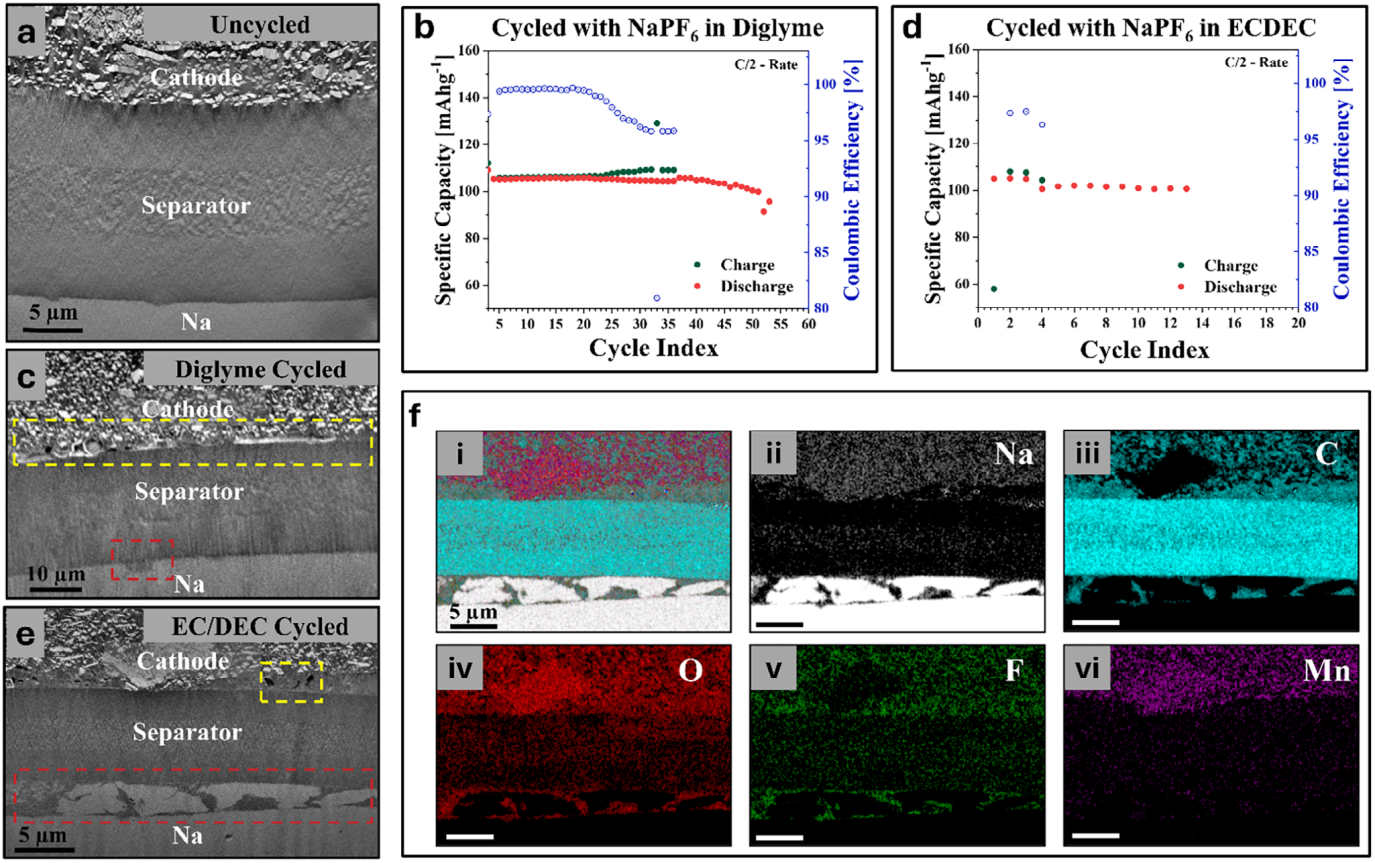
a) A cross‐sectional SEM image of an uncycled cell showing the three layers: cathode, separator, and Na metal anode. b) Plot of the specific capacity and Coulombic efficiency as a function of cycle number for a cell made with NaPF_6_ in diglyme. c) A cross‐sectional SEM image of a cell with NaPF_6_ in diglyme after 50 cycles. d) Plot of the specific capacity and Coulombic efficiency as a function of cycle number for a cell made with NaPF_6_ in EC/DEC. e) A cross‐sectional SEM image of a cell with NaPF_6_ in EC/DEC after 10 cycles. f) EDS maps of the region depicted in c representing the i. overlayed signal, ii. Na signal, iii. C signal, iv. O signal, v. F signal, and vi. Mn signal.

In **Figure** **2**, we present the low magnification scanning electron microscopy (SEM) images of the entire battery stacks and the respective energy dispersive X‐ray spectroscopy maps (EDX). As a baseline for comparison, uncycled cells were frozen, opened, and cross‐sectioned using cryo‐FIB. An example of the cross‐section of an uncycled cell is shown in Figure 2a. The cathode was a mix of Na_0.44_MnO_2_ particles suspended within a conductive carbon and polymer binder matrix. Particle distribution was relatively uniform, but some occasional large agglomerates were observed. These agglomerates often lead to increased surface roughness, resulting in variable contact between the cathode and the separator. The resultant gaps were filled with electrolyte, and the Na metal appeared uniform, as would be expected for an uncycled cell. Cells constructed with a diglyme‐based electrolyte were cycled from 2.0 to 3.8 V versus Na/Na^+^ at a rate of C/2 for 50 cycles following 3 formation cycles at a rate of C/20. One of these cells was also frozen, opened, and cross‐sectioned using cryo‐FIB. A plot of the Coulombic efficiency, charge capacity, and discharge capacity as a function of cycle number is shown in Figure 2b. Another version of this plot with expanded axes is shown in Figure S1a (Supporting Information). Performance is stable up until ≈25 cycles, at which point the coulombic efficiency begins to decrease, associated with an increase in the charge capacity. Between 35 and 40 cycles, the coulombic efficiency dramatically decreases, which is associated with a dramatic increase in charge capacity. At this point, the discharge capacity also begins to decline. In Figure S2 (Supporting Information), charge–discharge curves of the 10th and 50th cycles demonstrate the increase in charge capacity. d*Q*/d*V* plots of the same cycles (Figure S2b,d, Supporting Information) demonstrate that this increase in charge capacity is not associated with a real increase in energy storage but, instead, a large number of side reactions.

During charging, Na is removed from the cathode and plated onto the Na electrode. Na is stripped from the anode during discharge and intercalated into the cathode. For the cycled diglyme cells, shown in Figure 2c, the Na metal appears uniform, with no observable dendrites or “dead” Na. The only non‐uniformity present was a “‘pocket” likely containing SEI and some residual electrolyte (outlined in red in Figure 2c). This “pocket” may have formed during a dissolution step. The cathode‐separator interface shows more signs of degradation. As observed in uncycled cells, there are gaps between the cathode and the separator initially filled with electrolyte. Following cycling, voids are now observable (outlined in yellow in Figure 2c). These voids are attributed to electrolyte degradation and gas evolution during cycling, which has been preserved by the freezing process.

Figure 2d presents a plot of the Coulombic efficiency, charge capacity, and discharge capacity as a function of cycle number for a cell cycling in the EC/DEC electrolyte. Another version of this plot with expanded axes is shown in Figure S1b (Supporting Information). This cell was cycled 10 times at a rate of C/2 following 3 formation cycles at a rate of C/20. Like the diglyme cell, a large decrease in coulombic efficiency and associated increase in charge capacity is observed, occurring at cycle 5. Although this cell was cycled substantially less than the diglyme cell, the decrease in coulombic efficiency occurs at a proportional point in the cycle lifetime of the cells, suggesting that these cells are at an equivalent state of degradation. Unlike the cell cycled in diglyme electrolyte, the cell containing EC/DEC has significant evidence of non‐uniform Na plating/stripping, as shown in Figure 2e. Numerous domains of plated Na are observed accompanied by large volumes of SEI (outlined in red in Figure 2e). EDS maps of the Na, C, O, F, and Mn signals are shown in Figure 2f and help to identify the different material species present (Na metal, SEI, separator, and cathode). Like the diglyme cell, the cathode interface possesses regions of electrolyte in between the cathode and separator, now with voids trapped within (outlined in yellow in Figure 2e). However, the size and number of voids seems less than that observed in the diglyme cells.

**Figure 3:**
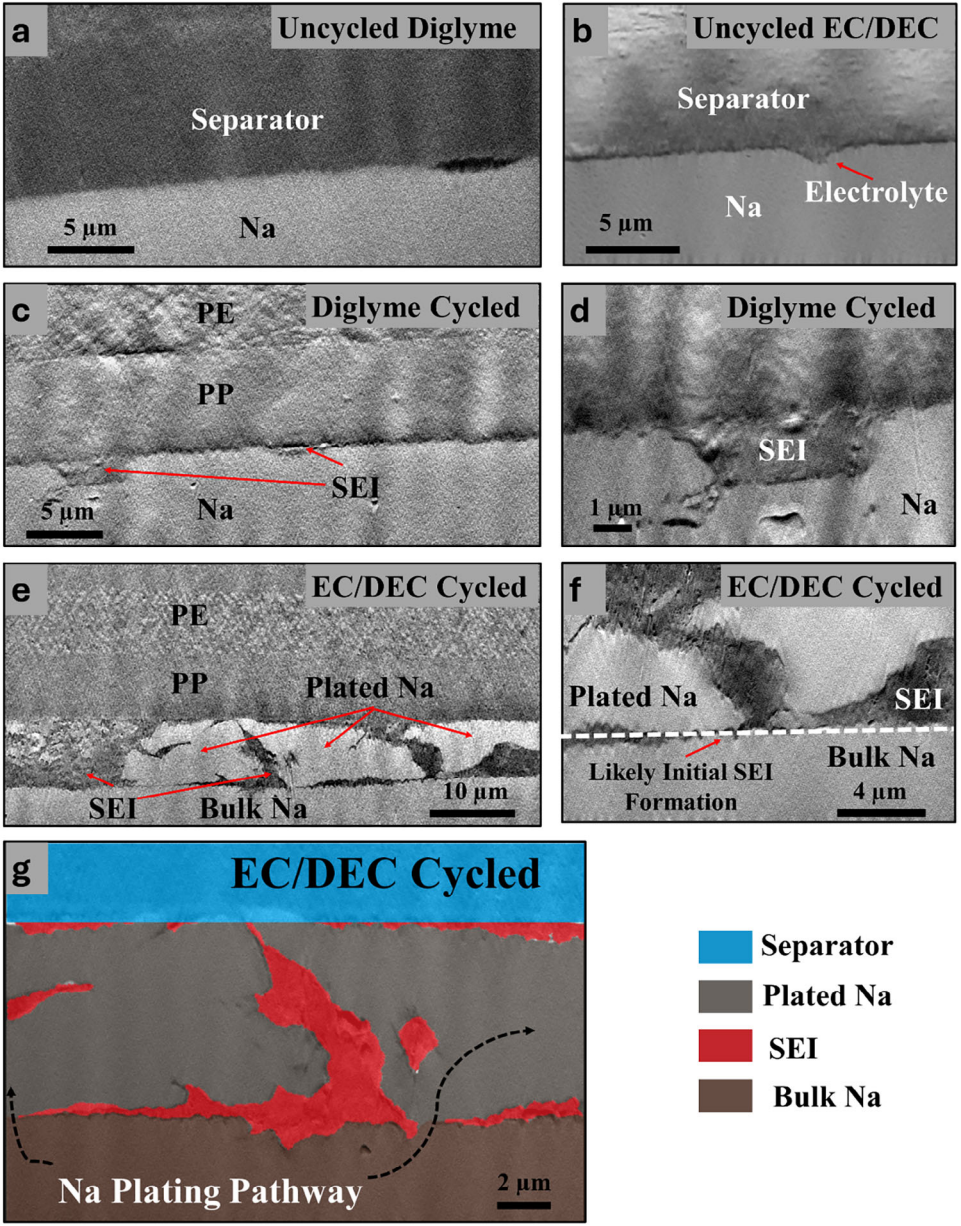
Cross‐sectional SEM images of the Na:separator interface of a) an uncycled cell with diglyme electrolyte, b) an uncycled cell with EC/DEC electrolyte, c) a cell after 50 cycles with diglyme electrolyte, d) a higher magnification images of plated Na regions from image c, e) a cell after 10 cycles with an EC/DEC electrolyte, f) a higher magnification image of the pocket of SEI from image e, and g) a false‐colored image of a region similar to f highlighting the location of plated Na and SEI.

**Figure** **3** presents high magnification cross‐sectional SEM images showing the Na metal electrode interface with the separator before and after cycling with either a carbonate‐ or diglyme‐based electrolyte. These images reveal critical insights into the morphology of plated Na and the formation of the SEI, significantly influencing SMBs performance and stability. Figure 3a,b shows the interface of uncycled cells with diglyme and EC/DEC electrolytes, respectively, providing a baseline for comparison. The interfaces are smooth and well‐defined, with only small gaps likely to form during cell construction. In Figure 3b, this gap is filled with electrolyte. There may be a thin contact SEI present; however, it cannot be clearly resolved by SEM. Figure 3c,e displays the interfaces after cycling with two different electrolyte systems. In Figure 3c, the cell cycled for 50 cycles with a diglyme‐based electrolyte exhibits a relatively uniform SEI layer, and the plated Na appears uniform. In fact, there is no clear distinction between bulk and plated Na. The morphology suggests that the diglyme electrolyte promotes stable SEI formation, likely due to its ability to form a flexible and passivating interphase, mitigating dendrite growth and uneven plating.^[^ ^6^ ^]^ In contrast, the EC/DEC electrolyte (Figure 3e) results in a more heterogeneous interface after only 10 cycles. The Na deposits exhibit uneven growth, with distinct regions of plated Na and irregular SEI layers. This uneven morphology can be attributed to the less stable nature of the SEI formed with carbonate‐based electrolytes, which are prone to cracking, dissolution, and localized growth due to their brittle and less elastic characteristics.

Figure 3d,f,g provides higher magnification views, revealing finer details of the interface. Figure 3d highlights a pocket of SEI from the diglyme‐based cell, likely formed within a pit that developed during stripping. However, across multiple cells probed, this does not occur frequently. In contrast, panels 3f and 3g reveal plated Na regions from the EC/DEC‐based cell. These images show significant SEI formation with irregular shapes. These regions of SEI appear both at the interface as well as within the electrodeposited Na. Figure 3g is false‐colored for easy identification of the plated Na and SEI. In these images, a clear threshold separates the initial bulk Na metal and subsequently deposited Na. A thin layer of SEI is observed separating the bulk from the deposited. In some regions, there is a small point with no SEI whatsoever, suggesting this is where the SEI first failed, allowing Na deposition to proceed above and around the existing SEI. This suggests that SEI forms during multiple cycles but cannot effectively passivate the surface, resulting in Na breaking through the SEI and growing above and around previously formed SEI. Comparing the diglyme and EC/DEC SEI thickness values shows order of magnitude difference, with ≈120 ± 40 nm thickness in Diglyme cases on average, and for EC/DEC SEI thickness values were up to 10 µm in regions where Na plating was completely absent, and then down to about 500nm in regions between the plated Na clusters and the bulk underlying Na electrode.

**Figure 4:**
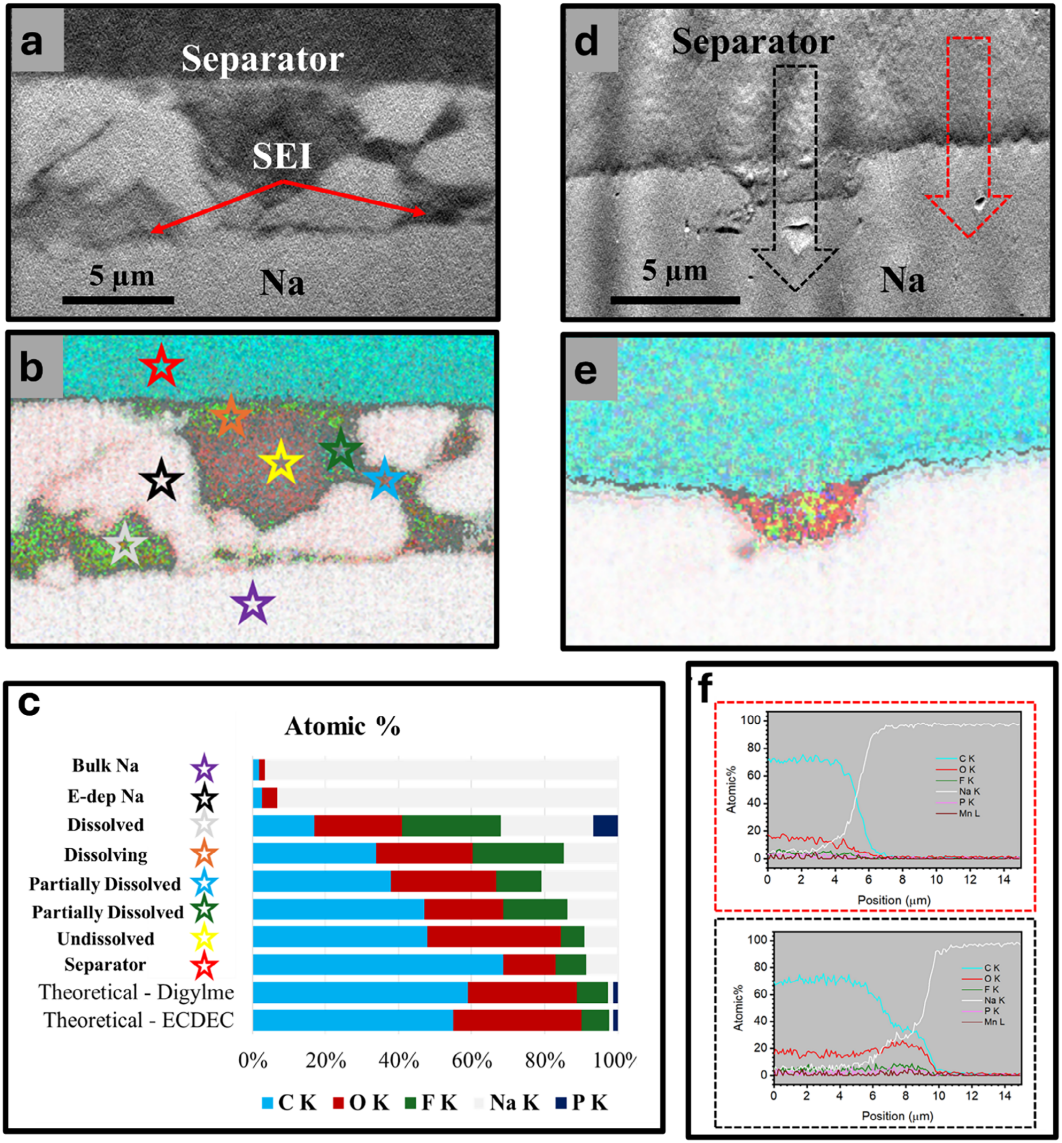
Cross‐sectional SEM images of the Na–separator interface of a cell after a) 10 cycles with NaPF6 in EC/DEC and d) 50 cycles with NaPF6 in diglyme, combined overlays of EDS maps of Na, C, O, F, P, and Mn for b) the region depicted in a, and e) the region depicted in d. c) A stacked bar graph showing the measured composition obtained from EDS at the spots marked with colored stars as well as the theoretical composition of the as‐prepared electrolytes. f) Line plots of composition as a function of distance; the plots outlined in red and black were measured across the regions marked with the red and black arrows in d, respectively.

**Figure** **4** uses EDX to map out the elemental composition of the Na‐separator interface after cycling in EC/DEC and diglyme‐based electrolytes, respectively. In Figure 4a (EC/DEC), SEM images show non‐uniform Na deposition and large pockets of what appears to be SEI. In contrast, Figure 4d (diglyme) shows uniform Na deposition with only a small pocket of what appears to be SEI present. The EDS maps in Figure 4b (EC/DEC) and 4e (diglyme) provide detailed elemental distributions across the Na interface. The presumed SEI in Figure 4b (EC/DEC) is rich in Na and F and poor in C, compared to the theoretical electrolyte composition. However, the composition is not consistent across all regions of SEI. Figure 4c quantitatively compares the composition at specific points within the SEI, as marked in Figure 4b. The stacked bar graph shows significant deviations between the measured and theoretical compositions of the as‐prepared electrolytes. The spot indicated by a yellow star in Figure 4b most closely resembles the electrolyte, though it still has increased Na content. This suggests that this region may either contain remanent electrolyte, and/or this region of SEI may be richer in carbonate/organo‐Na species. The regions indicated by the white and orange stars in Figure 4b have greatly reduced C and O signals and increased F content. This may be due to the tendency of the SEI to dissolve into the electrolyte. Other spots, such as those indicated by the blue and green spots in Figure 4b, have increased F content and reduced C content, though not to the same extent as the orange and white spots. This variation in SEI composition is attributed to varying degrees of SEI dissolution. These results suggest that the carbonate and organic species are more susceptible to dissolution in the SEI, leaving behind SEI that is rich in inorganic species like NaF. This dissolution also weakens the SEI's ability to passivate the surface, contributing to Na growing through and above the remaining SEI.

Figure 4f shows the compositional gradients in the diglyme‐based SEI through line plots measured across the regions indicated by arrows in Figure 4d for diglyme. These gradients suggest the presence of a thin, compact SEI layer composed primarily of Na, O, and C. These plots also confirm the pocket contains SEI based on the increased Na content relative to the expected electrolyte composition. Interestingly, there appears to be small amount of F signal present compared to O signal. Although NaF is often considered to be an ideal SEI component, it is possible Na_2_O may be preferred just as recent research has shown Li_2_O may be preferred to LiF.^[^ ^24^ ^]^ The SEI layer is too thin for accurate compositional analysis, but these results may suggest that Na_2_O may be a prominent SEI component, consistent with some prior literature.^[^ ^1^, ^25^ ^]^

**Figure 5:**
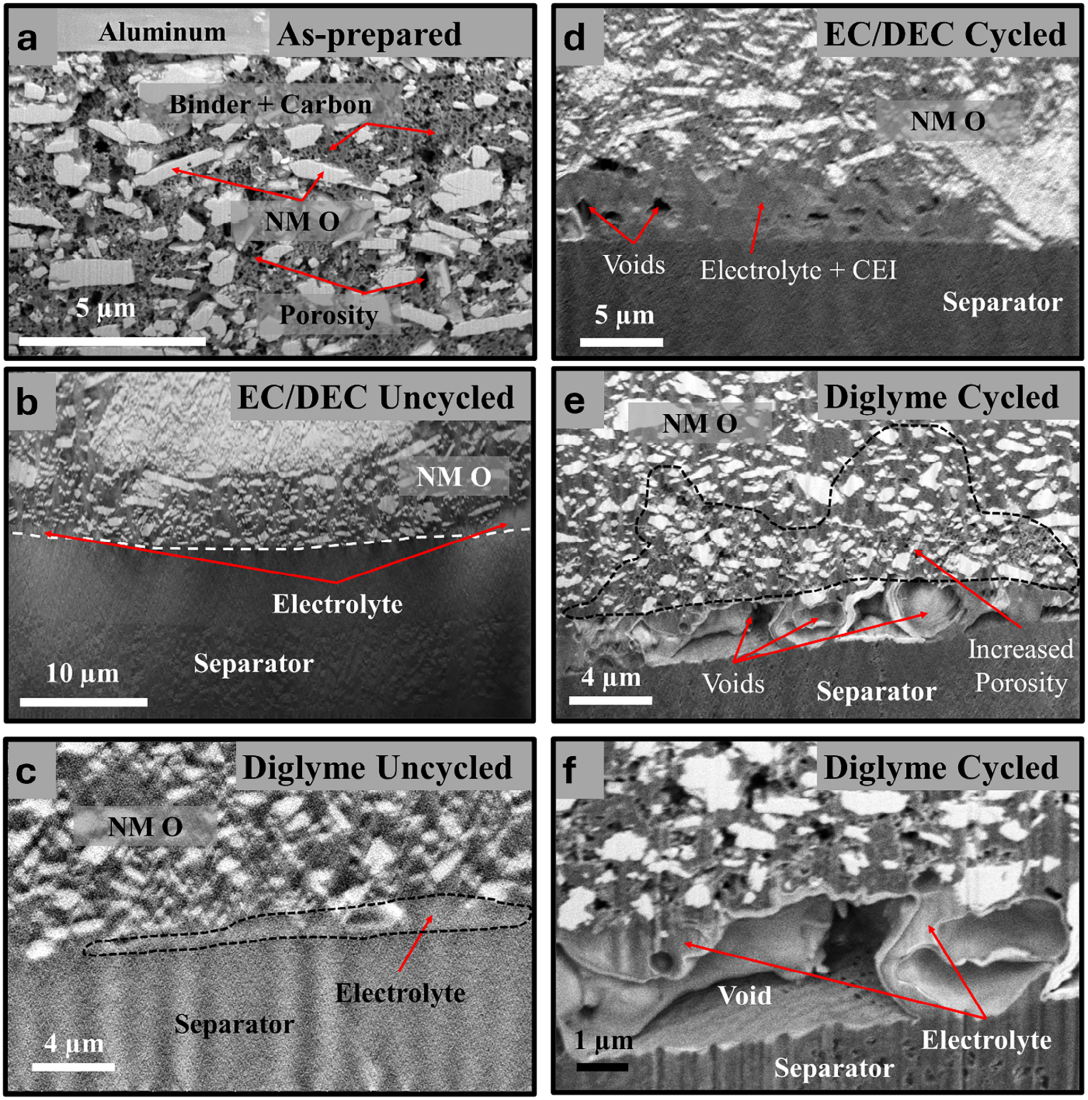
Cross‐sectional SEM images of a) an as‐prepared cathode before cell assembly, b) an uncycled EC/DEC cell, c) an uncycled diglyme cell, d) a cell after 10 cycles with EC/DEC electrolyte, e) a cell after 50 cycles with diglyme electrolyte, f) a higher magnification view of the cathode:separator interface seen in e.

**Figure** **5** shows the cathode–separator interface in detail through cross‐sectional SEM images that highlight the morphological evolution and the cathode microstructure during cycling in cells with diglyme‐based and carbonate‐based (EC/DEC) electrolytes. The figure emphasizes the role of electrolyte composition in influencing cathode porosity and interfacial stability, which are critical to the overall performance and longevity of SMBs. Figure 5a shows the dry as‐prepared cathode without any liquid electrolyte, serving as a baseline for comparison. The cathode contains Na_0.44_MnO_2_ particles distributed within a conductive carbon and polymer binder matrix. Many pores can be observed throughout the matrix, which allows the electrolyte to penetrate within the cathode structure. This image highlights the true porosity of the as‐prepared cathode before the liquid electrolyte fills the pores.

Figure 5b,c shows the cathode‐separator interfaces in uncycled cells prepared with EC/DEC and diglyme‐based electrolytes, respectively. The original porosity of the cathode has now been filled with electrolyte, and virtually no pores can be identified. Gaps between the cathode and the separator are also filled by electrolyte. Figure 5d,e illustrates the cathode–separator interface after cycling with EC/DEC and diglyme electrolytes, respectively. Large voids are observed at the interface in the diglyme‐based cell (Figure 5e), particularly in regions where the cathode's surface roughness prevents uniform contact with the separator. These voids, which were initially filled with electrolyte, likely result from electrolyte depletion, redistribution, and gas formation during cycling. The presence of voids suggests that while the diglyme electrolyte may stabilize the Na anode, it's lower oxidative stability could introduce challenges in maintaining intimate contact at the cathode‐separator interface, potentially increasing interfacial resistance. Additional SEM images of the cathode–separator interface can be seen in Figure S3 (Supporting Information). In contrast, the EC/DEC electrolyte (Figure 5d) exhibits less pronounced void formation at the interface but shows significant porosity throughout the cathode. This porosity arises from electrolyte decomposition and depletion during cycling, which appears to be more evenly distributed than in the diglyme case. The primary source for electrolyte depletion is likely the excessive growth of SEI at the anode, as was previously shown in Figure 3. Figure 5f provides a higher magnification image of the cathode‐separator interface in the diglyme‐based cell, revealing the intricate morphology of the voids, with some remaining electrolyte surrounding them. These voids are not explained by beam‐induced damage, as their size and shape are inconsistent with the small holes or blisters formed by excessive beam exposure (Figure S4, Supporting Information).

**Figure 6:**
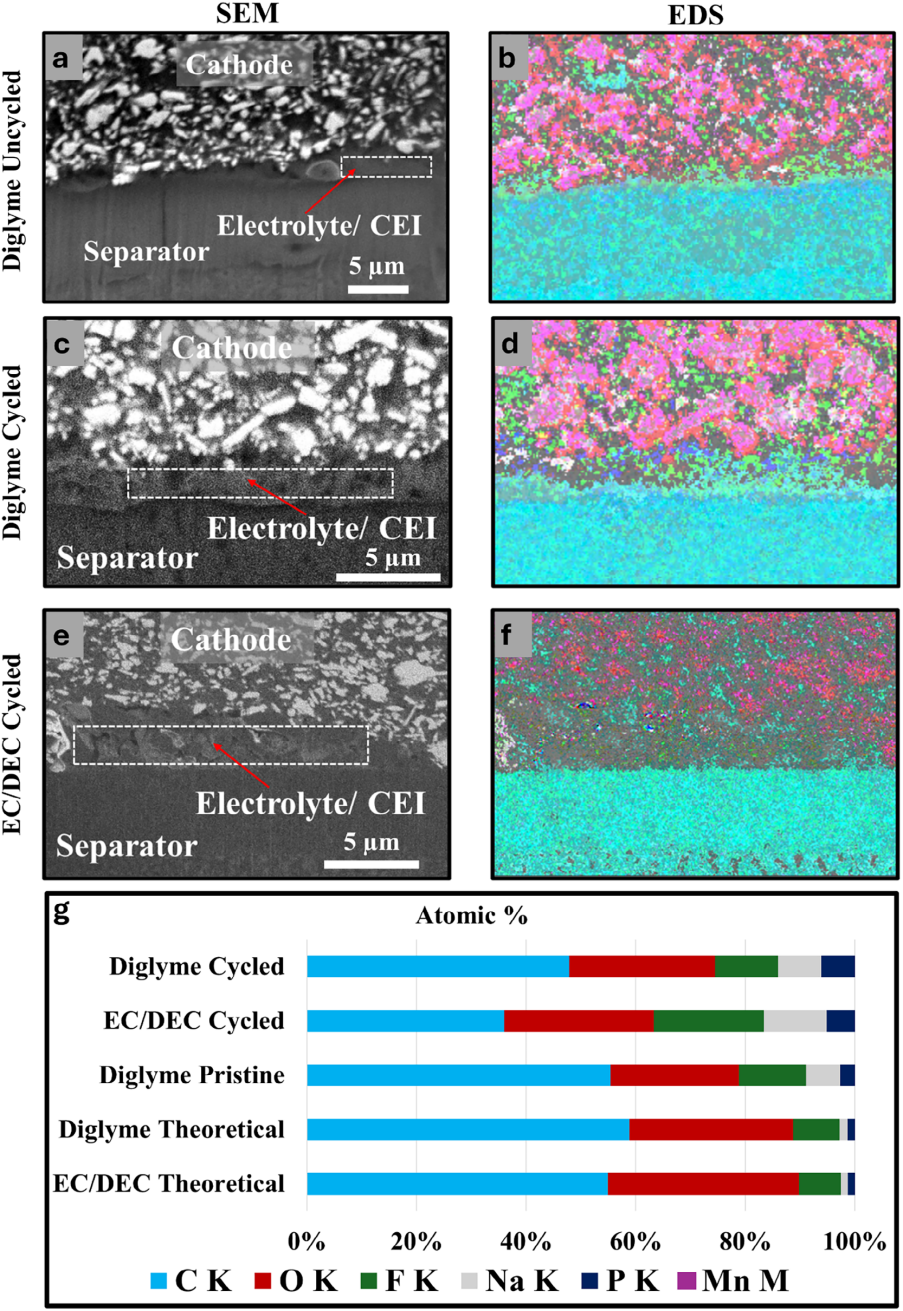
a) Cross‐sectional SEM image of the cathode:separator interface of an uncycled cell, b) the region shown in a overlayed with the EDS maps of Na, C, O, F, P and Mn, c) Cross‐sectional SEM image of the cathode:separator interface of a cell after 50 cycles with diglyme electrolyte, d) the region shown in c overlayed with the EDS maps of Na, C, O, F, P, and Mn, e) a cross‐sectional SEM image of the cathode:separator interface of a cell after 10 cycles with EC/DEC electrolyte, f) the region shown in c overlayed with the EDS maps of Na, C, O, F, P, and Mn, g) a stacked bar graph showing the composition of the theoretical electrolytes compared to the measured composition from the regions outlined in white in a, c, and e.

**Figure** **6** provides a detailed analysis of the elemental composition for the cathode‐separator interface and the cathode‐electrolyte interphase (CEI) for uncycled and cycled cells with diglyme‐ and carbonate‐based (EC/DEC) electrolytes using EDS. Figure 6a,b serves as a reference, presenting the cathode‐separator interface of an uncycled cell and the corresponding EDS maps. The EDS maps confirm the initial composition is consistent with theoretical electrolyte composition and establish the baseline for evaluating changes after cycling. Figure 6c,d shows the cathode‐separator interface and EDS maps for a cell cycled with a diglyme‐based electrolyte. The SEM image (Figure 6c) reveals the formation of voids and morphological irregularities at the interface, consistent with the electrolyte depletion and interfacial instability observed in previous figures. The EDS map (Figure 6d) shows a substantial presence of decomposition products, including Na, oxygen (O), fluorine (F), and carbon (C), indicative of CEI formation. The phosphorus (P) signal suggests residual electrolyte salts or their decomposition products, while the manganese (Mn) signal indicates minor cathode material dissolution or migration to the interface. Similarly, Figure 6e,f reveals the cathode–separator interface and EDS maps for a cell cycled with an EC/DEC‐based electrolyte. The SEM image (Figure 6e) highlights a more porous structure with a less distinct interface, suggesting a broader distribution of decomposition products within the CEI. The EDS map (Figure 6f) reveals a composition rich in Na, O, F, and C, with notable differences compared to the diglyme system. For instance, the EC/DEC electrolyte appears to produce a CEI with a higher concentration of fluoride species, likely reflecting the decomposition pathways of carbonate solvents and electrolyte salts.

Figure 6g provides a quantitative comparison of the CEI composition for each system. The stacked bar graph shows the measured compositions for the uncycled cells and the cycled cells with diglyme and EC/DEC electrolytes, alongside the theoretical compositions of the pristine electrolytes. The EC/DEC cell interface is enriched in Na and F species, indicative of increased electrolyte decomposition and formation of a CEI. Meanwhile, the interface of the diglyme cell much more closely resembles the composition of the electrolyte, with only a small increase in Na and F content, suggesting less CEI is formed.

**Figure 7:**
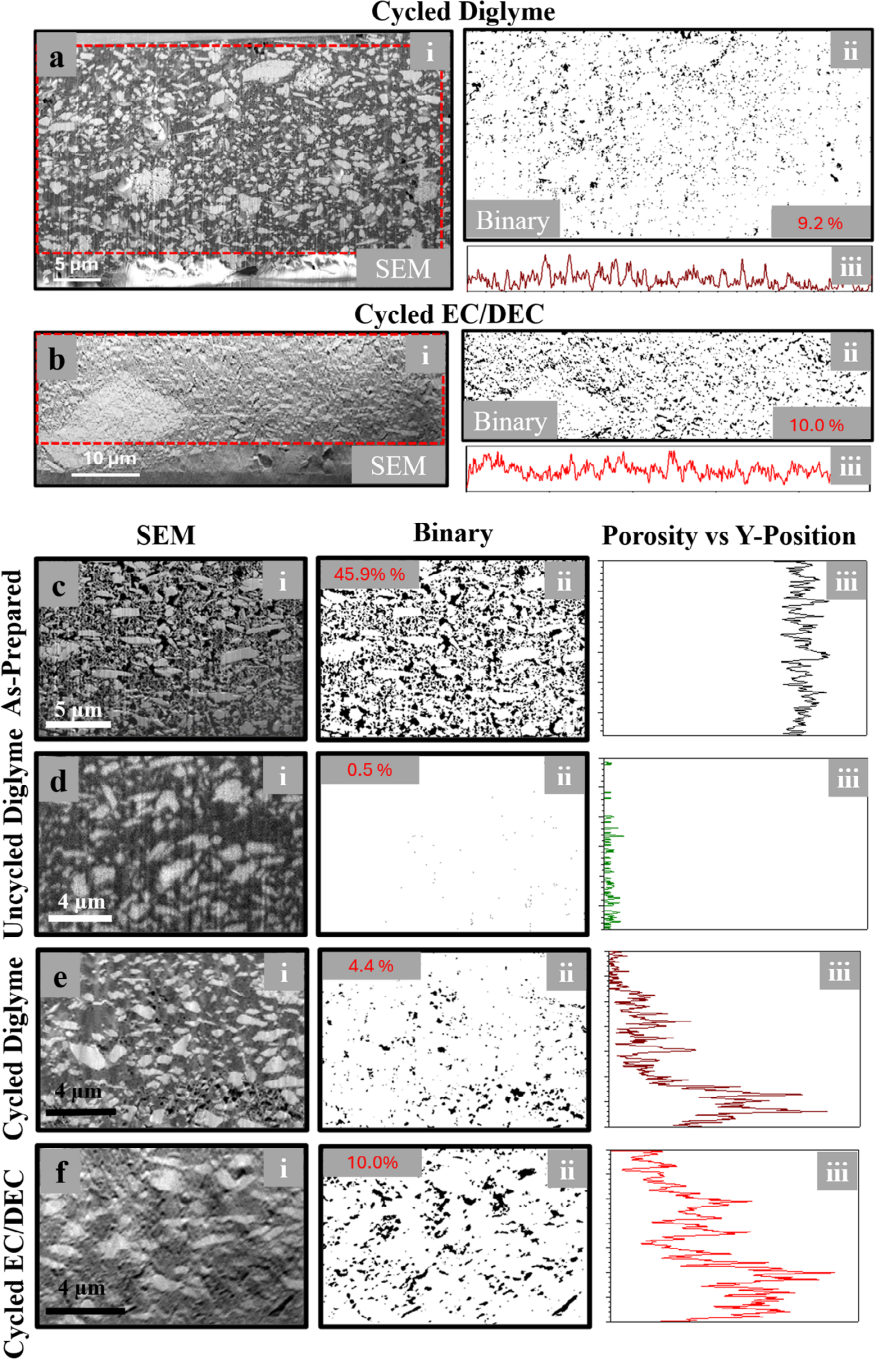
a,b) Cross‐sectional SEM images of the cathode (i), converted binary images of the region outline in red in a and b (ii), and a line profile of the porosity across the region imaged along the x‐axis (iii) for a cell after 50 cycles with a diglyme electrolyte and a cell after 10 cycles with an EC/DEC electrolyte, respectively. c–f) cross‐sectional SEM images of a region of the cathode (i), converted binary images of the same region shown in c–f (ii), and line profiles of the porosity across the region along the y‐axis (iii) for the as‐prepared cathode, uncycled cell, cell after 50 cycles in diglyme electrolyte, and cell after 10 cycles in EC/DEC electrolyte, respectively.

**Figure** **7** provides details of cathode porosity before and after cycling under different electrolyte conditions, using cross‐sectional SEM imaging, binary image processing, and porosity line profiles. Comparing porosity evolution across the diglyme and EC/DEC systems, as well as uncycled and as‐prepared cathodes, highlights how electrolyte choice and cycling influence cathode microstructure and interfacial behavior. Figure 7a,b examines the cathode after 50 cycles in a diglyme‐based electrolyte (Figure 7a) and 10 cycles in an EC/DEC‐based electrolyte (Figure 7b). The SEM images (i) show that the quantity and distribution of pores vary across the cathode. The binary images (ii) isolate the porosity, with black regions representing pores and white regions representing cathode material. Line profiles (iii) show porosity as a function of the x‐position across the imaged region. Both cells have relatively consistent porosity as a function of x‐position, with the EC/DEC cell possessing higher overall porosity compared to the diglyme system. It is worth noting that porosity tended to increase around the edges of NMO particles, especially large agglomerates, as shown in Figure S5 (Supporting Information).

Figure 7c,d provides baseline comparisons for the as‐prepared cathode (Figure 7c) and the uncycled cell (Figure 7d). In the as‐prepared cathode (Figure 7c), the SEM image (i) reveals a high porosity structure inherent to the cathode fabrication process, which is uniformly distributed, as confirmed by the binary image (ii) and the line profile (iii) along the y‐axis. This maximum porosity is significantly reduced in the pristine cell (Figure 7d), where electrolyte filling during cell assembly almost eliminates void spaces. The line profile in (Figure 7d‐iii) demonstrates a relatively uniform porosity gradient along the y‐axis, reflecting the absence of porosity variance in the uncycled cell. Figure 7e,f zoom in on higher magnification regions of the cathodes cycled in diglyme (Figure 7e) and EC/DEC (Figure 7f) electrolytes. The SEM images (i) and corresponding binary images (ii) highlight localized differences in porosity near the cathode‐separator interface. The line profiles (iii) along the y‐axis illustrate more distinct trends: the diglyme‐based cell (Figure 7e‐iii) shows a steep increase in porosity near the interface, corresponding to electrolyte depletion and void formation. Conversely, the EC/DEC‐based cell (Figure 7f‐iii) demonstrates a more gradual porosity gradient, consistent with the pervasive electrolyte decomposition and porous CEI formation observed in other analyses. Additional images and binary analyses for other regions are shown in Figure S6 (Supporting Information).

The as‐prepared Na_0_._44_MnO_2_ cathode exhibits an initial porosity of 9.2%, representing the baseline microstructural void fraction of the composite. Upon electrolyte infiltration, the apparent porosity remains nearly unchanged at 10.0%, indicating that the pore network is almost entirely filled with liquid electrolyte, leaving only residual unfilled volume. After electrochemical cycling, however, the carbonate‐based EC/DEC cell shows a five‐fold increase in porosity to 45.9%, signifying severe electrolyte depletion and gas evolution within the cathode matrix. In contrast, the ether‐based diglyme cell exhibits a minimal rise to 4.4%, corresponding to only half the initial porosity of the as‐prepared electrode, demonstrating that the structure remains largely intact. The uncycled reference sample shows an extremely low porosity of 0.5%, confirming that the voids observed in cycled cells originate from electrochemical reactions rather than sample preparation. Furthermore, localized measurements near the cathode–separator interface reveal that the EC/DEC system develops interfacial voids with a local area fraction of 10.0%, more than double that found in the diglyme cell under identical cycling conditions. Overall, the EC/DEC electrolyte increases the effective cathode porosity by approximately 36 percentage points relative to the diglyme system, a nearly ten‐fold enhancement in void volume, quantitatively underscoring the greater instability and gas generation associated with carbonate decomposition.

Overall, Figure 7 reveals that the diglyme electrolyte leads to a non‐uniform porosity profile with pronounced void formation near the cathode‐separator interface, likely due to gas generation and local electrolyte depletion. The EC/DEC electrolyte, while promoting a more even porosity distribution, results in higher overall porosity and a more porous cathode structure. These findings align with observations from earlier figures, underscoring the critical influence of electrolyte composition on cathode microstructure evolution and interfacial stability during cycling. The differences in porosity profiles provide further insight into the challenges associated with each electrolyte system and highlight the need for strategies to minimize void formation and reduce electrolyte depletion during prolonged cycling.

**Figure 8:**
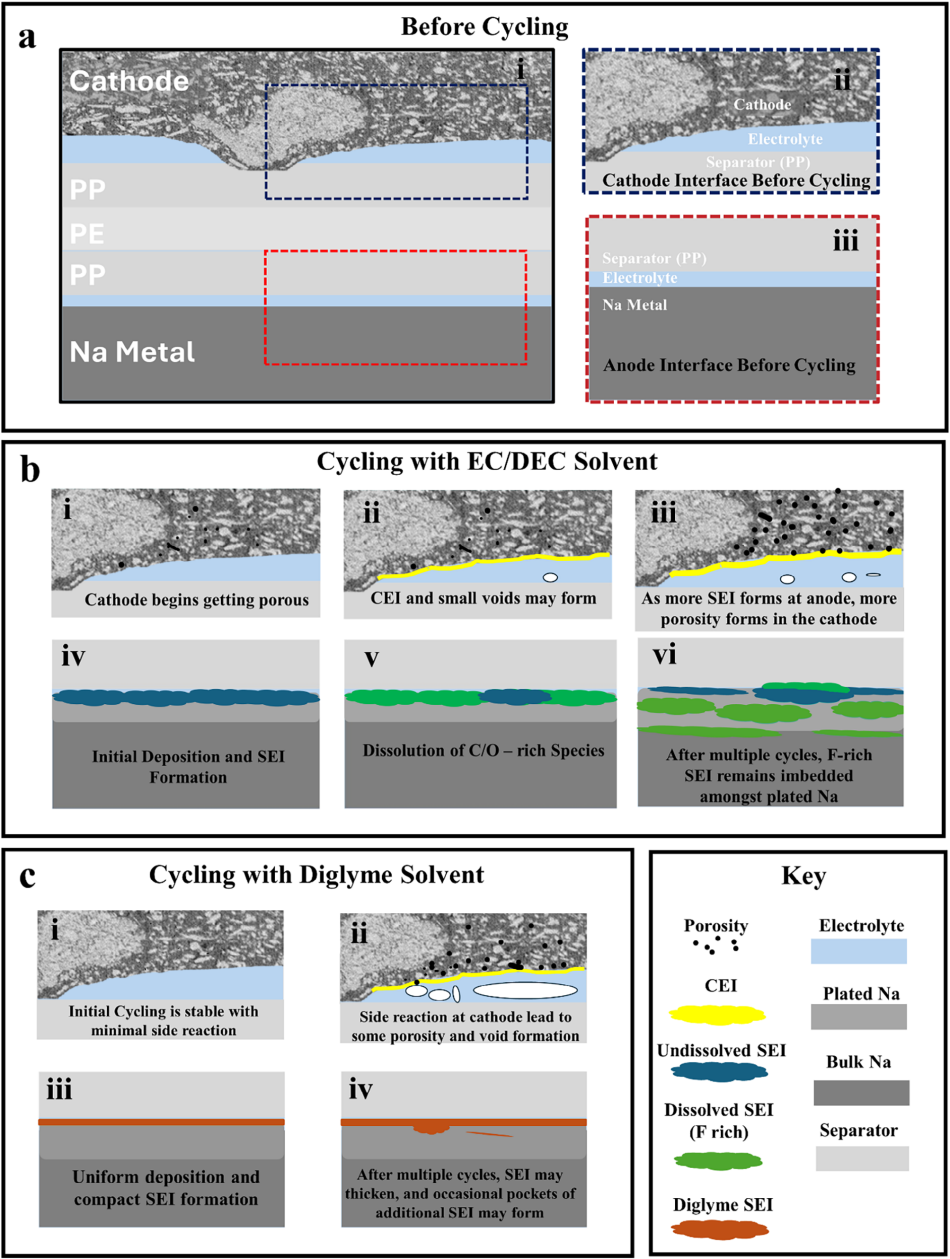
A schematic explaining the interface evolution at the cathode and anode for both a cell cycled with a diglyme‐based electrolyte and an EC/DEC‐based electrolyte. a) The entire cell (i) and a zoomed‐in view of the cathode (ii) and anode (iii) before cycling. b) The evolution of the cathode–separator interface (i–iii) and the anode‐separator interface (iv–vi) while cycling with an EC/DEC‐based electrolyte; i/iv. Initial charge cycle, ii/v. following discharge cycle, iii/vi. after extended cycling. c) The evolution of the cathode‐separator interface (i, iii) and the anode‐separator interface (ii, iv) while cycling with a diglyme‐based electrolyte; i/iii. Initial charge cycle, ii/iv. after extended cycling.

**Figure** **8** provides a schematic representation of the interfacial evolution for both electrolyte systems. For the EC/DEC system (Figure 8b), an initially thick SEI forms and then partially dissolves over time, leading to instability and irregular Na plating. Extended cycling exacerbates these effects, resulting in a porous and fragmented SEI. This leaves points at which Na may grow through the SEI, ultimately growing around and trapping some regions of SEI. Simultaneously, this excessive SEI growth leads to depletion of the electrolyte which is observed as increased porosity within the cathode. Ultimately, it is the poor stability of the anode interface which results in the EC/DEC cell's quick failure.

In contrast, the diglyme system (Figure 8c) forms a thinner but more cohesive SEI that remains stable over extended cycling. Although an occasional pit may form, the Na deposition is overwhelmingly uniform, contributing to improved capacity retention and cycle lifetime. This difference underscores the ability of diglyme to support uniform Na plating and mitigate side reactions at the anode. However, the lower oxidative stability of diglyme results in degradation of the electrolyte at the cathode, resulting in electrolyte depletion and void formation. The porosity observed within the cathode is not quite as severe given there is not the same excessive SEI formation. Still, the void formation occurring at higher cycle numbers leads to a similar reduction in performance, though much later in the cell's lifetime. This highlights the importance of characterizing and evaluating the entire battery cell as all components contribute to the overall cell's performance.

To place these observations in context, the contrasting degradation pathways observed for EC/DEC and diglyme electrolytes illustrate two distinct regimes of interfacial control in SMBs. In carbonate systems, anode instability dominates, leading to early failure through electrolyte depletion and heterogeneous SEI growth. In ether systems, the anode remains passivated, shifting the failure locus to the cathode–electrolyte interphase, where oxidative degradation and gas evolution ultimately compromise contact. This mechanistic distinction emphasizes that long‐term stability in Na batteries requires concurrent stabilization of both anode and cathode interfaces, rather than optimization of either in isolation.

It is noted that the Na_0_._44_MnO_2_ cathodes employed here (≈3 mg cm^−^^2^) represent a relatively low areal loading designed to facilitate uniform freezing and FIB cross‐sectioning. Higher‐load electrodes would likely exacerbate cathode‐side electrolyte depletion due to longer Na⁺ diffusion paths and greater local current densities. Nonetheless, the mechanistic trends identified, anode‐dominated failure in EC/DEC and cathode‐dominated degradation in diglyme, are intrinsic to solvent stability rather than geometric parameters, and are therefore expected to persist in thicker electrodes.

## 3 Conclusion

This study not only visualizes but also mechanistically delineates the solvent‐dependent degradation regimes in SMBs. Through full‐cell cryo‐FIB and EDX analyses, we reveal that carbonate electrolytes (EC/DEC) lead to an anode‐limited failure pathway, dominated by unstable SEI formation and continuous electrolyte consumption, whereas ether‐based diglyme electrolytes exhibit a cathode‐limited failure mechanism, governed by oxidative electrolyte decomposition and interfacial void formation. These findings demonstrate that the electrochemical stability of SMBs is determined not by a single electrode, but by the dynamic balance of both anodic and cathodic interfacial energetics.

Quantitative analysis further strengthens this mechanistic framework. The SEI in diglyme‐based cells remains thin (≈180 nm) and uniform, while that formed in EC/DEC systems is approximately three times thicker and structurally heterogeneous. Correspondingly, binary image analysis of cathode microstructure shows a ≈4% void fraction for diglyme electrolytes compared to ≈10% for carbonate systems, indicating that electrolyte depletion and gas evolution proceed through distinct pathways in the two solvent environments. These quantitative metrics not only enhance the rigor of our conclusions but also establish benchmark parameters for future computational and experimental modeling of interfacial degradation.

The results confirm that diglyme electrolytes promote uniform SEI formation at the Na metal anode, suppressing dendrite nucleation and supporting stable cycling, yet suffer from localized void generation at the cathode–separator interface due to limited oxidative stability. In contrast, EC/DEC electrolytes produce a brittle, heterogeneous SEI that fails to passivate the anode effectively, resulting in uneven Na deposition, excessive SEI growth, and accelerated degradation across both electrodes.

Overall, both electrolyte systems exhibit increased cathode porosity with cycling, approximately twofold higher in carbonate cells, but through different mechanisms: diglyme degradation remains spatially confined near the interface, whereas carbonate breakdown leads to widespread porous CEI formation throughout the cathode. While cryo‐FIB and SEM/EDX provide compelling mesoscale evidence for these processes, complementary cryo‐TEM or cryo‐STEM studies could further validate the nanoscale structure and chemistry of the SEI and CEI.

By enabling direct, intact cross‐sectional imaging of both electrodes within the same frozen cell, the full‐cell cryo‐FIB approach established here provides a powerful blueprint for multiscale analysis of liquid‐electrolyte batteries. This methodology allows spatial correlation between electrolyte composition and interfacial degradation, offering a direct route to designing next‐generation electrolytes that simultaneously stabilize both anode and cathode interfaces, minimize void formation, and extend the lifetime of practical SMBs.

## 4 Experimental Section

### Materials

Na hexafluorophosphate (NaPF6, 99+%, Alfa Aesar) was used as received from the manufacturer. Diethylene glycol dimethyl ether (anhydrous, 99.5%, Sigma‐Aldrich) was transferred into clean secondary containers with Activated 0.3 nm molecular sieves for further use. 0.3 nm Molecular sieve beads (Supelco, Sigma‐Aldrich) were first activated at 200 °C for 15 h before transfer into an Ar‐filled glovebox (Bruker, H_2_O < 0.1ppm, O_2_ < 0.1ppm) for further use. Na cubes (99.9%, Sigma‐Aldrich) were used for rolling out Na electrodes, the mineral oil from cubes was removed using hexane solvent, and then the oxidized surface was removed before rolling electrodes. Na manganese oxide (Na0.44MnO_2_, NANOMYTE) was obtained from NEI corporation. PVDF binder was obtained from MTI (HSV900 PVDF binder). The Celgard 2325 Trilayer membrane was dried in a vacuum oven for 48 hours before transferring into the glovebox for coin cell fabrication.

### Experimental Methods for Electrolyte/Electrode Preparation—Preparation of Electrolytes

The solvents dried in molecular sieves (0.3 nm, Supelco, Sigma‐Aldrich) were used for electrolyte preparation. All the electrolytes were made with 1 m concentration of NaPF_6_ as the salt. The corresponding solvents and salt were mixed and stirred for 2 h to ensure salt dissolution. Then, the electrolyte solution was dried using activated molecular sieves overnight before using them for cell fabrication.

### Experimental Methods for Electrolyte/Electrode Preparation—Preparation of Electrodes

The Na electrodes were prepared through a rolling‐folding process. Typically, the Na electrode cubes are rolled to a flat sheet of (≈200 µm thickness) inside the glovebox and then cut into circular electrodes (1/2 in.) for further use. An NMO (Na0.44MnO2) cathode was fabricated following reference.^[^ ^26^ ^]^ To obtain working cathodes, the material was mixed with a conductive carbon (Super P) and poly(vinylidene fluoride) (PVDF) binder at a weight ratio of 80:10:5 in 1‐methyl‐2‐pyrrolidone (NMP) solvent. The slurry was then coated onto an Al foil (0.5 in.). The electrodes were vacuum dried at 120 °C overnight and cut into disks. The average mass loading of NMO electrodes was around 3.0 mg cm^−2^. NMO versus Na/Na+, 2.0–3.8 V voltage range was used for all full‐cell cycling.

### Experimental Methods for Electrochemical Characterization—Cell Fabrication

Symmetrical (Na||Na) cells, full cells (Na||NMO), and half‐cells (Cu||Na) were cycled in CR2032 coin cells with an Arbin multichannel battery testing system. All coin cells were assembled in a glovebox (<0.1 ppm H_2_O, <0.1 ppm O_2_), and electrochemical tests were conducted using a coin cell setup. Celgard 2325 separator was used in all cells. An excess electrolyte (>5 drops) was used to flood the cell and provide sufficient wetting of the separator. The electrochemical results presented are an average of triplicate cells to ensure the reproducibility of the results. Prior to cell cycling, all coin cells were left to rest for 12 h to form a native SEI.

### Experimental Methods for Cryogenic Sample Preparation and Imaging—Sample Preparation and Transfer

Coin cells were submerged into liquid nitrogen and reached equilibrium in <30 s. Coin cells typically remained under liquid nitrogen for 15–30 min prior to the cell being opened. Slush nitrogen could be used instead of liquid nitrogen, though liquid nitrogen provided adequate cooling for coin cells made with carbonate‐based electrolytes was found, which have a high freezing point (ethylene carbonate is solid at room temperature). Using pliers, the cell was opened under liquid nitrogen as shown in Figure 1. The pliers were pre‐cooled in liquid nitrogen to prevent transferring heat to the cell. The center stack containing both electrodes and the separator was removed. In many cases, one of the spacers was still attached to one of the electrodes and served as the bottom of the stack. The stack was added to a small container filled with liquid nitrogen and transferred to the Leica vacuum‐cryo manipulation loading station (VCM) where it was loaded onto the sample holder. The sample was oriented such that the NMO cathode was on top. The thickness of the cathode and current collector is approximately 50 µm, which is much thinner than the approximately 500 µm thick Na metal, making it much quicker to mill through in order to expose all layers of the cell. The VCM, VCT, and sample holder were baked out overnight to remove adsorbed moisture prior to cooling. The VCM, VCT, and sample holder were cooled to −196 °C prior to transferring the sample. Once the sample was loaded onto the sample holder, it was retracted into the VCT and pumped to vacuum. The VCT was pre‐cooled to approximately −170 °C. The VCT was docked onto the Scios‐FIB‐SEM which was fitted with a compatible dock and a Leica cryo‐stage. The cryo‐stage is cooled by many copper bands attached to a liquid nitrogen dewar. The cryo‐stage was pre‐cooled to approximately −155 °C and remained between −145 and −155 °C throughout the experiment. Once docked, the sample holder was inserted and secured in the cryo‐stage.

### Experimental Methods for Cryogenic Sample Preparation and Imaging—Cryogenic Focused Ion Beam Milling

Given the sample had an aluminum current collector on top, no Pt was deposited as the aluminum was conductive and thick enough to provide adequate protection to the layers below. For bulk mills, the ion beam was operated at 30 kV with a beam current of 65 nA. A high beam current was necessary to allow for wide and deep mills that would expose the full extent of the NMO:Separator:Na stack. To fully probe the interfaces between the separator and both electrodes, trenches that were at least 50 µm wide, 100 µm long, and 100 µm deep were required. After bulk milling, cleaning cross‐sections were performed. The first cleaning cross‐section was typically performed at 30 nA with subsequent cleanings at 15 and 7 nA. In some cases, a final clean at 5 nA was performed. This combination of cleaning cross‐section was found to be the most time efficient while also providing a nice facet for imaging.

### Experimental Methods for Cryogenic Sample Preparation and Imaging—Scanning Electron Microscopy and Energy Dispersive X‐Ray Spectroscopy

Samples were imaged in a ThermoFisher Scios Dual‐Beam FIB‐SEM using an Eberhart Thornley secondary electron detector (ETD) and an in‐lens backscattered electron detector (T1). Samples were imaged at 2 and 5 kV with currents of 0.4 and 1.6 nA. As many of the samples experience curtaining artifacts from the FIB process, the images were filtered for vertical stripes using the method shown in Figure S7 (Supporting Information). EDS was performed with a windowless 30 mm^2^ Ultradry detector at 5 and 10 kV. Though this is a lower voltage than typical for EDS, it was sufficient for collecting the relevant chemical information. Higher voltages were avoided as they lead to increased sample damage, even at cryo‐temperatures. 10 kV was originally selected to allow detection of the Mn K edge, however, the observed damage, even at just 10 kV, lead to 5 kV being the primary operating voltage. Although the Mn K edge could no longer be probed when operating at 5 kV, the Mn L edges proved sufficient. At least 50 frames were collected to have adequate counts. Data processing was performed with ThermoFisher Pathfinder software.

## Conflict of Interest

The authors declare no conflict of interest.

## Supporting information



Supporting Information

## Data Availability

The data that support the findings of this study are available from the corresponding author upon reasonable request.
