# Supplementary material for: Revealing Cycling‐Induced Evolution of Intact Sodium Metal Battery Interfaces Using Cryo‐Focused Ion Beam Cross‐Sectioning and Electron Microscopy
Source: Small. 2025 Dec 15;22(7):e08531. doi: 10.1002/smll.202508531 (PMC12862447; doi:10.1002/smll.202508531)
Supplement: Supplementary file 1 — Supporting Information [file SMLL-22-e08531-s001.docx]

Supplementary Information

Revealing Cycling-Induced Evolution of Intact Sodium Metal Battery Interfaces Using Cryo-Focused Ion Beam Cross-sectioning and Electron Microscopy

*Kevin C. Matthews^1^, Rinish R. Vaidyula^3^, C. Buddie Mullin^1,3, 4^, Jamie H. Warner^1, 2, *^*

^1^ Materials Science and Engineering Program and Texas Materials Institute, The University of Texas at Austin, 204 East Dean Keeton Street, Austin, Texas 78712, United States

^2^ Walker Department of Mechanical Engineering, The University of Texas at Austin, 204 East Dean Keeton Street, Austin, Texas 78712, United States

^3^ Department of Chemistry, The University of Texas at Austin, Austin, Texas 78712, United States.

^4^ John J. McKetta Department of Chemical Engineering, The University of Texas at Austin, Austin, Texas 78712, United States

**Email:* [*Jamie.warner@austin.utexas.edu*](mailto:Jamie.warner@austin.utexas.edu)*;*


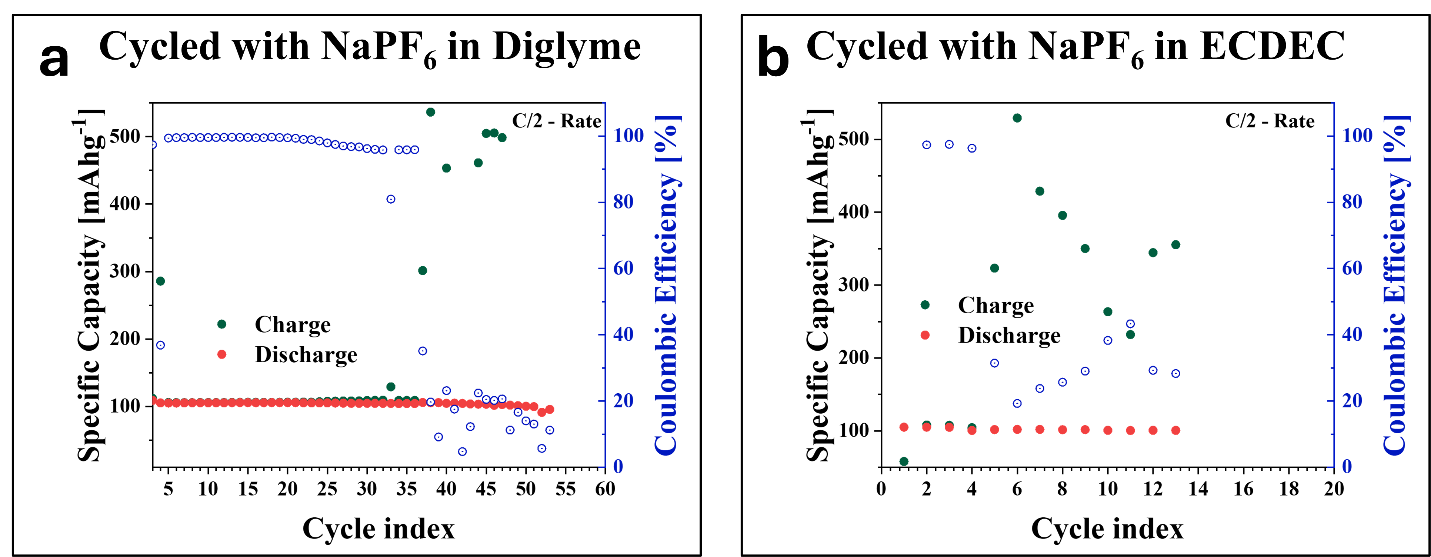


**Figure S1** – a) Plot of the Specific Capacity and Coulombic efficiency as a function of cycle number for a cell made with NaPF_6_ in diglyme electrolyte, b) Plot of the Specific Capacity and Coulombic efficiency as a function of cycle number for a cell made with NaPF_6_ in EC/DEC electrolyte. Axes limits are extended to allow all data points to be visible.


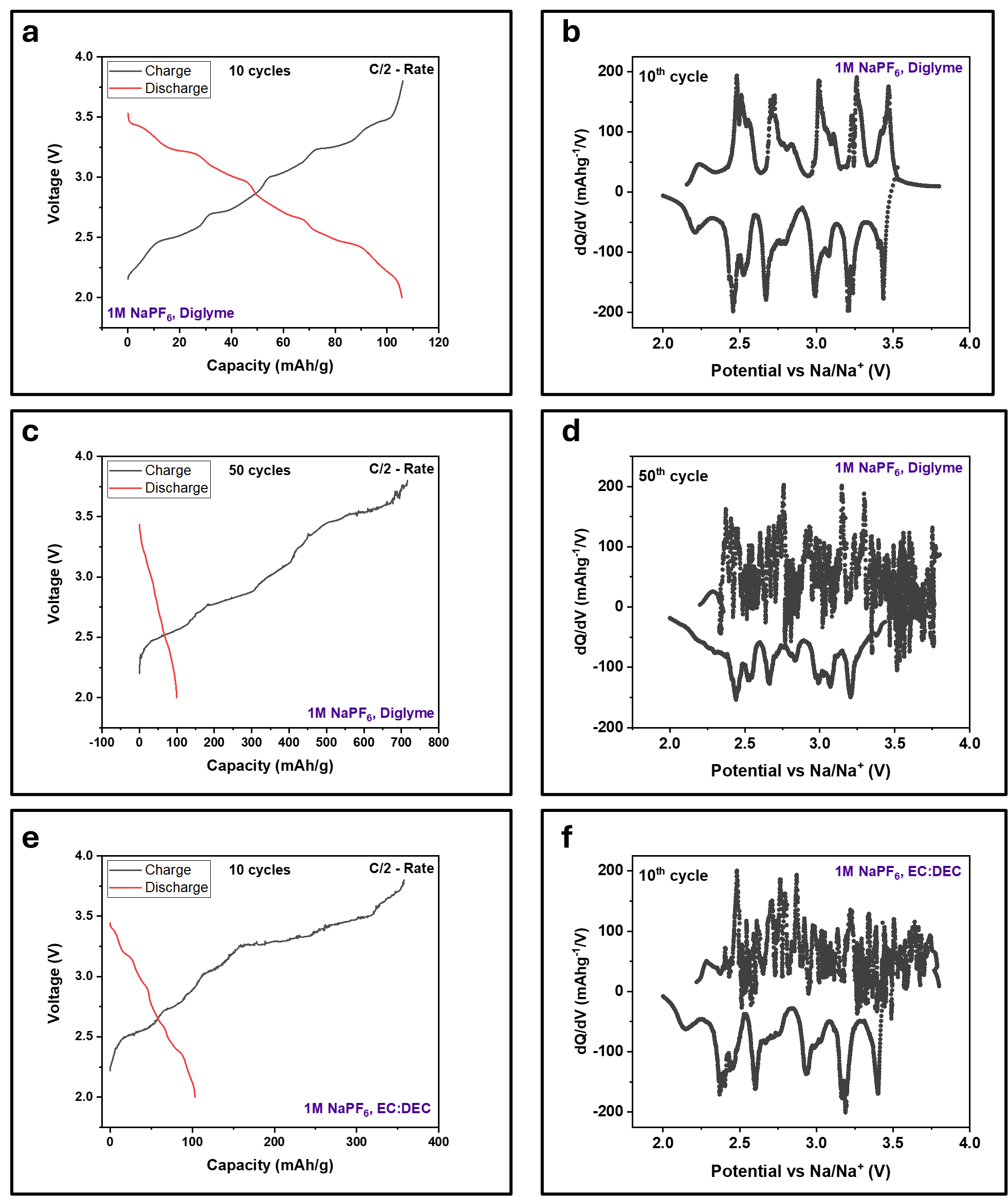


**Figure S2** – Charge-Discharge plots and corresponding dQ/dV plots for the 10^th^ (a, b) and 50^th^ (c, d) cycle of a diglyme cell and the 10^th^ (e, f) cycle of an EC/DEC cell


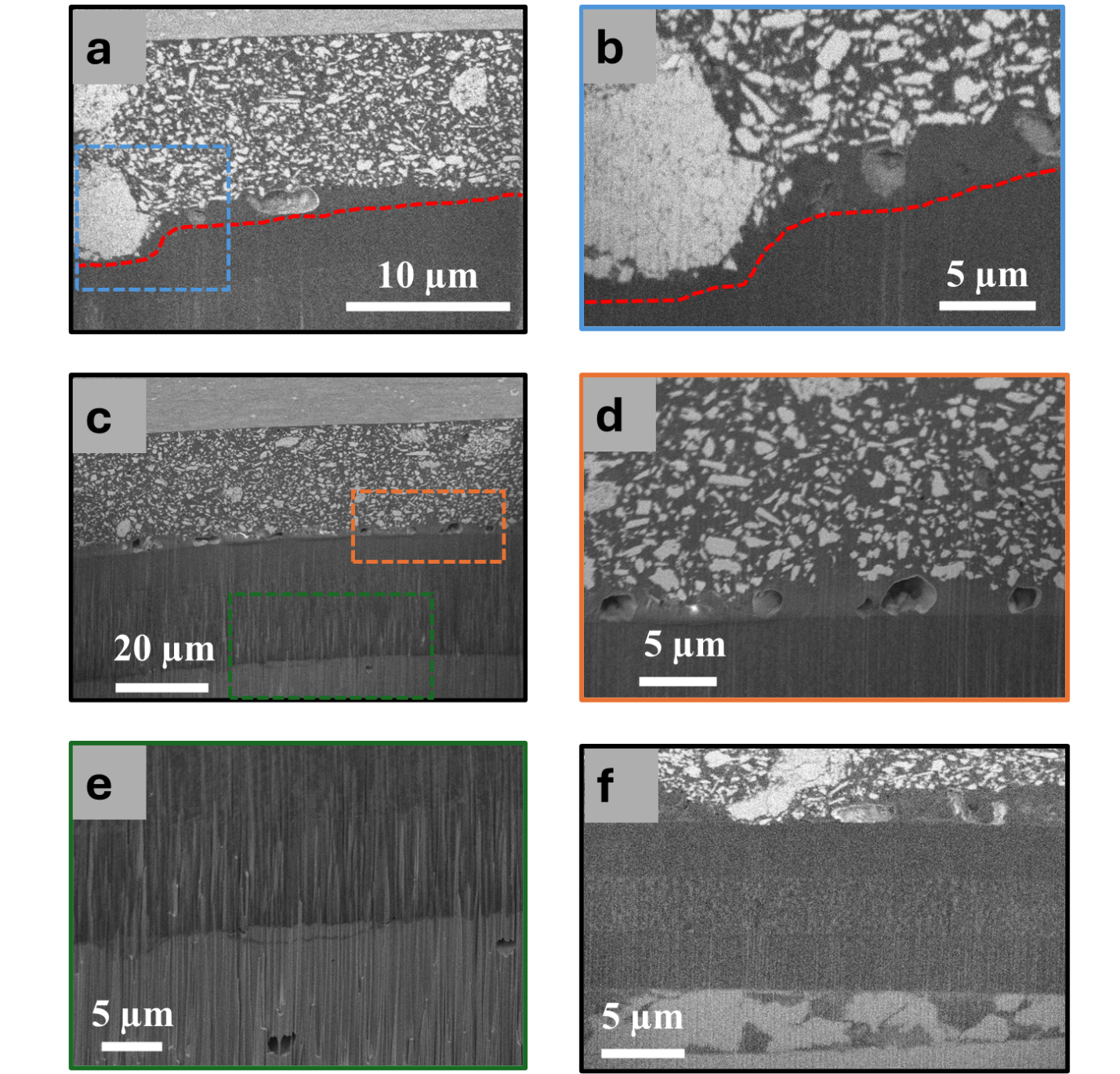


**Figure S3** – Cross-sectional SEM images of a) a cycled diglyme cell highlighting the distortion of the separator by a large agglomerate of Na_0.44_MnO_2_ particles, b) a zoomed-in high magnification image of the region outlined in blue in **a**, c) another region in a cycled diglyme cell, d) a zoomed-in high magnification image of the region outlined in orange in **c**, e) a zoomed-in high magnification image of the region outline in green in **c**, and f) a cycled EC/DEC cell.


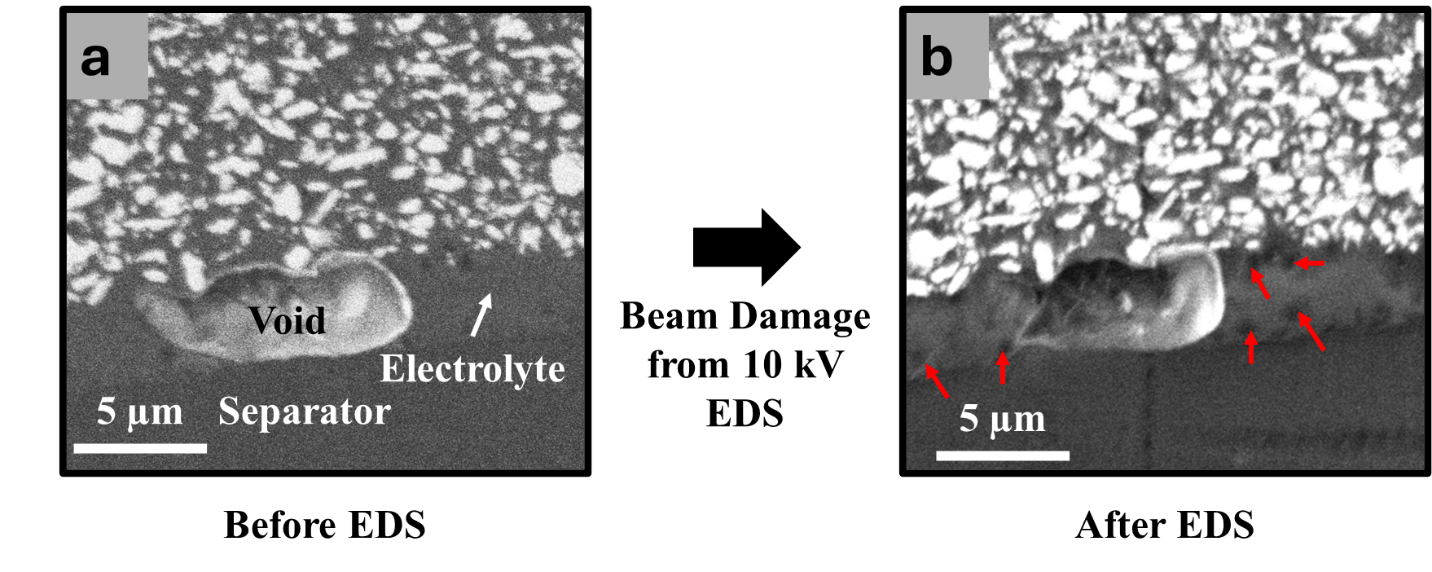


**Figure S4** – Beam-induced damage to the electrolyte at the cathode-separator interface before (a) and after (b) 10 kV EDS mapping. Red arrows in **b** point to small beam-induced holes which differ significantly from the voids intrinsic to the cells.


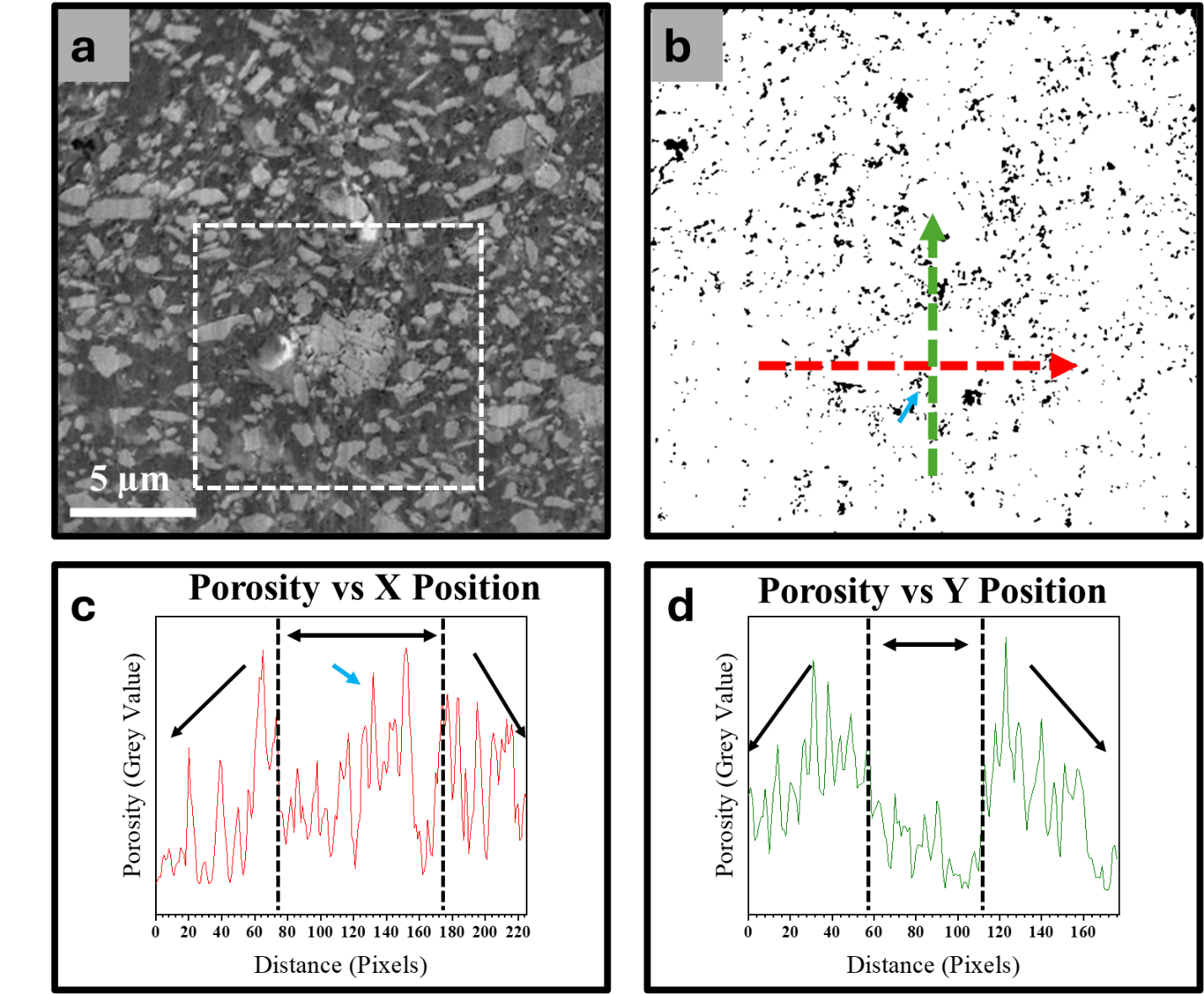


**Figure S5** – a) A cross-sectional SEM image of the cathode after 50 cycles with a diglyme-based electrolyte; the white box outlines the agglomerate analyzed in subsequent panels. b) The image in **a** binarized; the red and green arrows signify the directions of the plots presented in **c** and **d**. c) A line plot of the porosity as a function of X position across the agglomerate; the blue arrow corresponds to the porosity highlighted by the blue arrow in b. d) A line plot of the porosity as a function of Y position across the agglomerate. For both **c** and **d**, the dotted black lines mark the edges of the agglomerate.


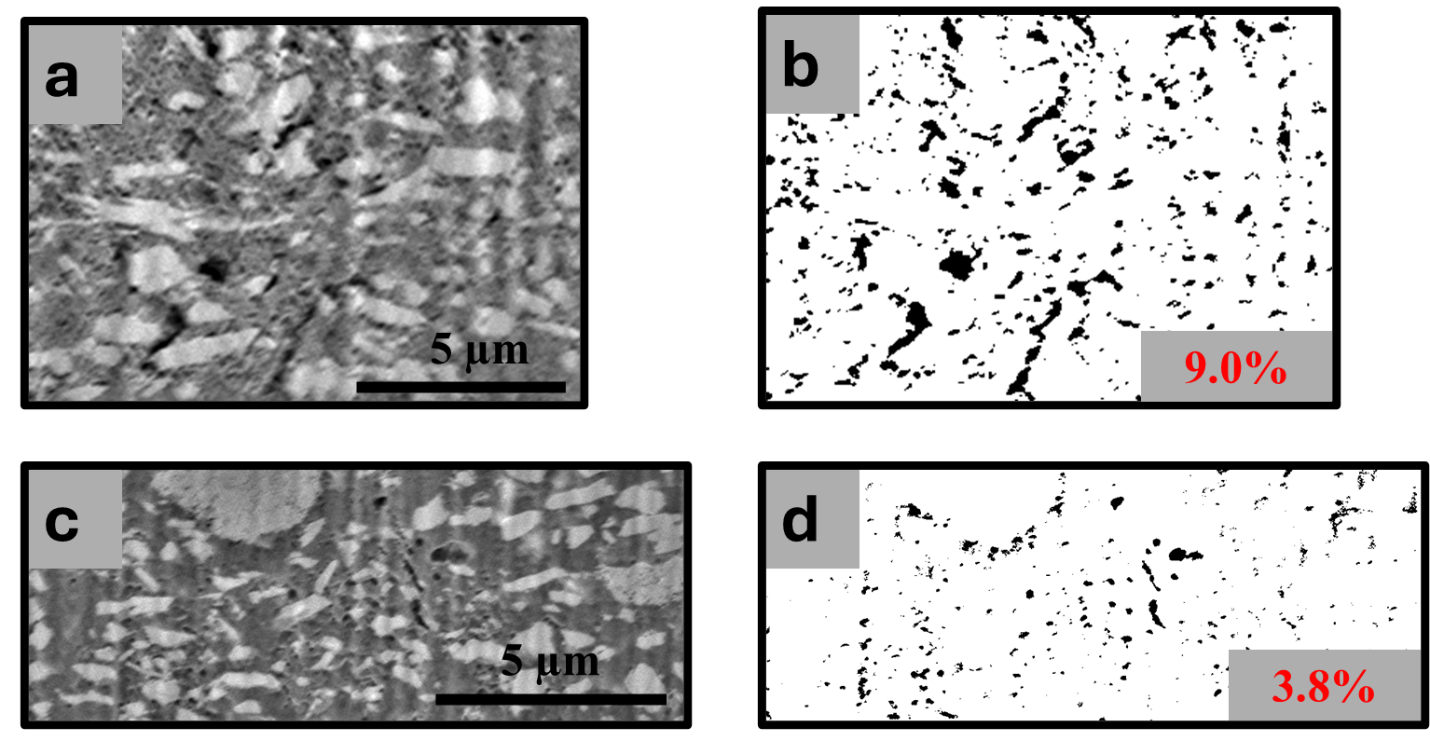


**Figure S6** – Additional cross-sectional SEM images of a cycled EC/DEC (a) and diglyme (c) cells with corresponding binary images (b and d, respectively) highlighting the porosity of the cathode.


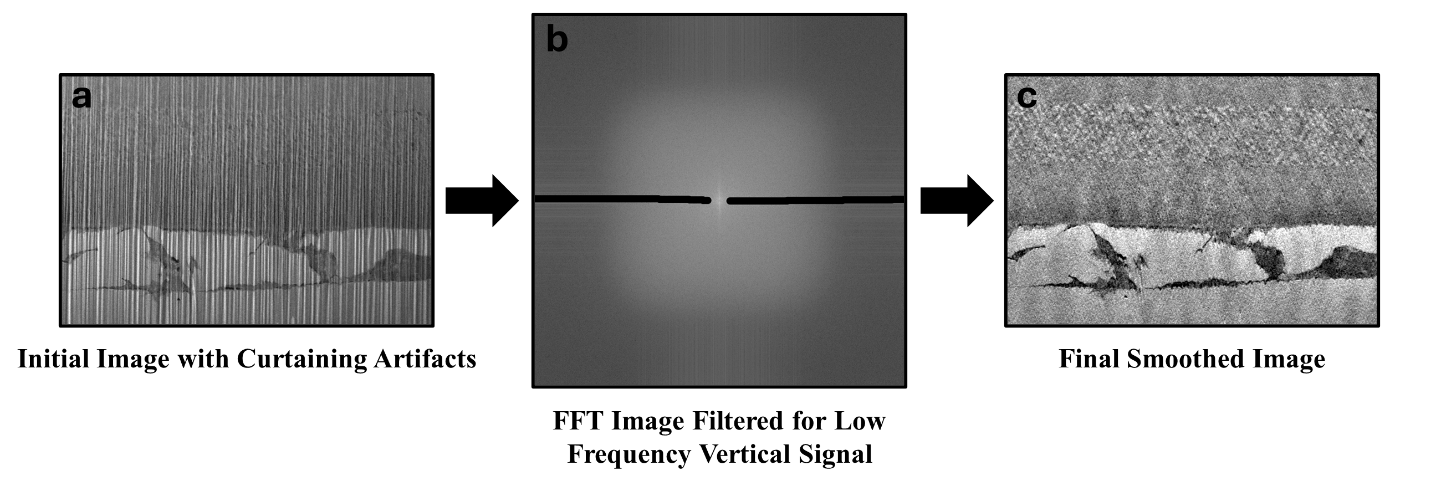


**Figure S7** – Process for removing curtaining artifacts from an initial SEM image (a) by modifying the FFT (b) to remove low frequency vertical signal and produce a final smoothed image (c).
